# “What Not” Detectors Help the Brain See in Depth

**DOI:** 10.1016/j.cub.2017.03.074

**Published:** 2017-05-22

**Authors:** Nuno R. Goncalves, Andrew E. Welchman

**Affiliations:** 1 Department of Psychology, University of Cambridge, Downing Street, Cambridge CB2 3EB, UK

**Keywords:** depth perception, convolutional neural network, da Vinci stereopsis, 3D vision, wallpaper illusion, binocular disparity

## Abstract

Binocular stereopsis is one of the primary cues for three-dimensional (3D) vision in species ranging from insects to primates. Understanding how the brain extracts depth from two different retinal images represents a tractable challenge in sensory neuroscience that has so far evaded full explanation. Central to current thinking is the idea that the brain needs to identify matching features in the two retinal images (i.e., solving the “stereoscopic correspondence problem”) so that the depth of objects in the world can be triangulated. Although intuitive, this approach fails to account for key physiological and perceptual observations. We show that formulating the problem to identify “correct matches” is suboptimal and propose an alternative, based on optimal information encoding, that mixes disparity detection with “proscription”: exploiting dissimilar features to provide evidence against unlikely interpretations. We demonstrate the role of these “what not” responses in a neural network optimized to extract depth in natural images. The network combines information for and against the likely depth structure of the viewed scene, naturally reproducing key characteristics of both neural responses and perceptual interpretations. We capture the encoding and readout computations of the network in simple analytical form and derive a binocular likelihood model that provides a unified account of long-standing puzzles in 3D vision at the physiological and perceptual levels. We suggest that marrying detection with proscription provides an effective coding strategy for sensory estimation that may be useful for diverse feature domains (e.g., motion) and multisensory integration.

## Highlights

• The brain uses “what not” detectors to facilitate 3D vision
• Binocular mismatches are used to drive suppression of incompatible depths
• Proscription accounts for depth perception without binocular correspondence
• A simple analytical model captures perceptual and neural responses

## Teaser

Goncalves and Welchman show that long-standing puzzles for the physiology and perception of 3D vision are explained by the brain’s use of “what not” detectors. These facilitate stereopsis by providing evidence against interpretations that are incompatible with the true structure of the scene.

## Introduction

Geometry dictates that a three-dimensional (3D) object viewed from the two eyes will (1) project features to different positions on the two retinae and (2) render certain portions visible to only one eye due to occlusion at the object’s contours [1]. Computational [2, 3, 4] and neurophysiological [5] investigations over the past 50 years have focused almost exclusively on positional differences (1), as partial occlusions (2) are regarded as excessively under-constrained. Under this intuitive approach, by registering the positional difference of the same feature in the two eyes (binocular disparity), the brain could triangulate to infer the object’s 3D structure. Thus, while the genesis of binocular information lies in image *differences*, current understanding at the computational and neural levels stresses the centrality of identifying *similarities* between the eyes to extract depth.

**Figure 1:**
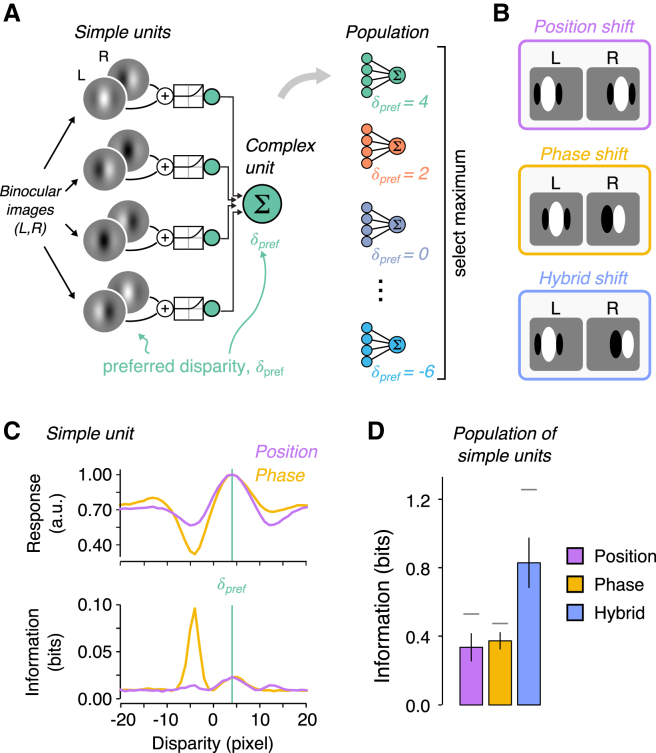
Disparity Encoding and Shannon Information (A) The canonical disparity energy model. Simple and complex units have the same preferred disparity, $\delta_{pref}$. (B) Simple cells encode disparity using differences in receptive field: position (position disparity), structure (phase disparity), or both (hybrid). (C) Mean response of model simple units to 100,000 stereograms (top) and the corresponding Shannon information (bottom). Pink versus yellow series contrast pure position versus phase (*π* / 2) encoding, both with $\delta_{pref}$ = 4. Considering units between pure position and pure phase encoding produces a graceful morphing in the shapes of the curves. (D) Shannon information for a small population (N = 5) of simple units with position, phase, or hybrid sensors. (Computing Shannon information for larger populations was computationally prohibitive.) Error bars show SD over 1,000 populations with randomly distributed phase and/or position shifts. Horizontal lines depict the upper limit on information determined by a population with uniformly spaced units.

Within this framework, the fundamental challenge of stereopsis is described as solving the “correspondence problem” [2, 3, 4] whereby images of the same real-world feature are matched between the eyes. This is problematic because of “false matches,” i.e., correspondences that conflate signals originating from different locations in 3D space. The principal means of identifying corresponding features is to consider a range of potential disparities and select the offset that maximizes similarity between the eyes. This is captured computationally by the peak local cross-correlation. How might this be achieved by the brain? Current understanding is provided by the disparity energy model of V1 neurons [6, 7, 8], in which binocular simple cells with disparity preference, $\delta_{pref}$, are combined by a complex cell preferring the same disparity (Figure 1A). Using a population of cells with different $\delta_{pref}$, the brain could select the most active neuron to estimate depth.

However, from the perspective of finding correct matches, it is puzzling that many V1 neurons sense different things in the two eyes [9, 10, 11]. In particular, while binocular neurons can have receptive fields offset in location (position disparity), they often have different receptive field profiles in the two eyes (phase disparity) (Figure 1B). The surprising implication is that phase neurons respond maximally to images that do not relate to a single physical feature in the world [12]. What are such responses for?

Here we suggest that V1 neurons should be understood as using a coding strategy designed to reduce uncertainty about the depth of the viewed scene. This involves the brain using both similar and dissimilar image features to infer depth. We show that long-standing puzzles in binocular vision at the physiological and perceptual levels can be understood by mixing feature detection with “proscription.” Specifically, by sensing *dissimilar* features, the brain gains valuable information that drives suppression of unlikely interpretations of the scene. Our approach explains challenges to the standard treatment of disparity (1) and, importantly, also accounts for partial occlusions (2) that have long evaded explanation because of their incompatibility with registering depth based on peak cross-correlation.

## Results

We start by considering known properties of binocular neurons from a statistical perspective [13], to demonstrate that properties that have long seemed puzzling in fact suggest optimal coding. Position-disparity units (Figure 1B, purple) are easily understood from the traditional perspective: a viewed object will project its features to different locations on the two retinae, so a binocular unit could simply offset the receptive field location for the two eyes. Phase-disparity units (Figure 1B, orange), by contrast, have a different receptive field structure in the two eyes. This means they respond best to stimulation that could not originate from a single physical feature in the world. We contrasted phase and position encoding by computing Shannon information [13] as a function of stimulus disparity (see STAR Methods), where simple units were modeled as linear filters followed by a rectified squaring non-linearity [6]. Because of the larger change in firing of the phase units, they provide more information about the viewed stimulus than position units (Figure 1C). Importantly, the peak information provided by a phase unit is not at the traditionally labeled $\delta_{pref}$ (i.e., peak firing rate), meaning that the disparity energy model’s architecture (Figure 1A) of collating signals from units with the same $\delta_{pref}$ is likely to be suboptimal. We then examined encoding in a small population of simple units with position, phase, or hybrid receptive fields. We found that hybrid encoding (i.e., combined phase and position shifts; Figure 1B) conveys more information than either pure phase or position encoding (Figure 1D). This suggests that the abundance of hybrid selectivity in V1 neurons [9, 10, 11] may relate to optimal encoding.

**Figure 2:**
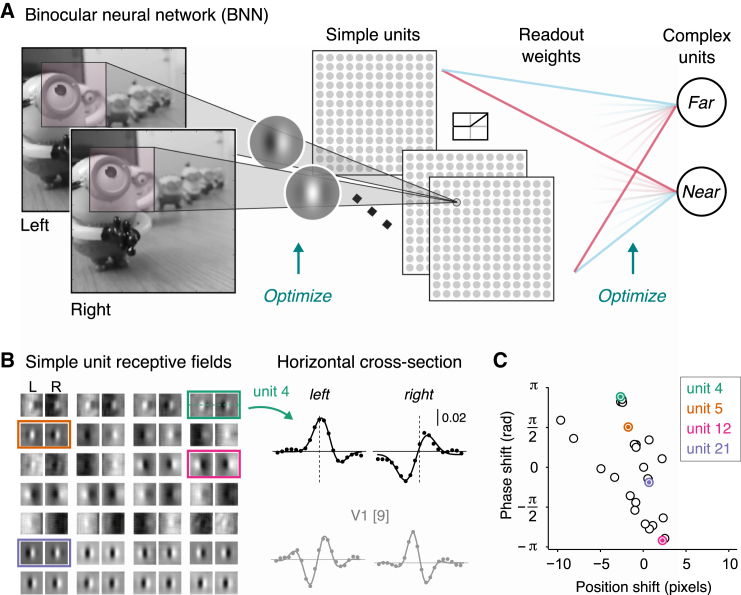
The Binocular Neural Network (A) Network architecture: left and right images are filtered by simple units (28 binocular convolutional kernels), linearly rectified, and then read out by two output units. The form of the (1) receptive fields and (2) readout weights was determined through back-propagation optimization on near versus far depth discrimination using patches from stereoscopic natural images (from [14]). The network learned 21,254 parameters through exposure to 32,300 image pairs. (B) The BNN’s optimized receptive fields resembled Gabor functions (mean explained variance by fitting Gabors to the 28 binocular receptive fields was R^2^ = 0.95, SD = 0.049) and V1 receptive fields [9]. (C) Summary of position and phase encoding by the simple units; representative units from (B) are indicated in colors. Note that very few units show pure position or phase offsets. See also Figure S1 and Figure S2.

To test the idea that V1 neurons are optimized to extract binocular information, we developed a model system shaped by exposure to natural images. We implemented a binocular neural network (BNN; Figure 2A) consisting of a bank of linear filters followed by a rectifying non-linearity. These “simple units” were then pooled and read out by an output layer (“complex units”). The binocular receptive fields and readout weights were optimized by supervised training on a near-versus-far depth discrimination task using patches from natural images (Figure S1). Thereafter, the BNN classified depth in novel images with high accuracy (A = 99.23%).

### Optimization with Natural Images Produces Units that Resemble Neurons

**Figure 3:**
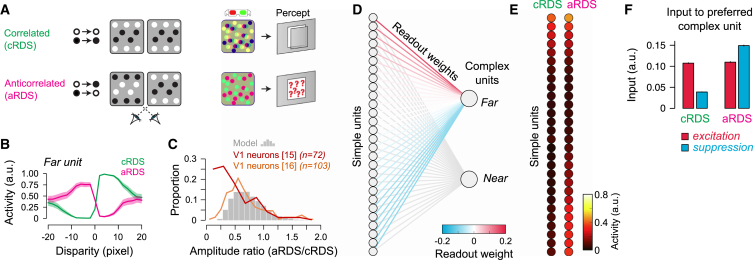
BNN Response to Correlated and Anticorrelated Random-Dot Stereograms (A) Cartoons of correlated (cRDS, green) and anticorrelated (aRDS, pink) dot patterns with red-green anaglyph demonstrations. (B) Complex unit’s disparity tuning curve for cRDS versus aRDS; shaded area shows CI_95%_. (C) Distribution of amplitude ratios for cRDS versus aRDS for the BNN (gray histogram; 5,000 resamples) and macaque V1 neurons. Amplitude ratios were determined based on Gabor fits (average explained variance, R^2^ = 0.945). (D) Representation of the weighted readout of the simple units. Units are ordered by their readout weight, with far-preferred units at the top. (E) Mean activity for simple units in response to cRDS and aRDS. (F) Summary of excitatory (red) and suppressive (blue) drive to the output units for cRDS versus aRDS. This represents the sum of the weighted simple unit activity split into the excitatory (positive weights) and suppressive (negative weights) components. Error bars (barely visible) indicate CI_95%_. See also Figure S3.

The optimized structure of the BNN resembled known properties of simple and complex neurons in three main respects. First, simple units’ receptive fields were approximated by Gabor functions (Figure 2B) that exploit hybrid encoding (Figure 2C; Figure S2) [9, 10, 11] with physiologically plausible spatial frequency bandwidths (mean = 2.3 octaves). Second, like V1 neurons, the BNN supported excellent decoding of depth in correlated random dot stereogram (cRDS) stimuli (Figure 3A) (A = 99.93%; CI_95_% = 99.87%, 99.98%) that are traditionally used in the laboratory, despite being trained exclusively on natural images. Third, we tested the BNN with anticorrelated stimuli (aRDS) where disparity is depicted such that a dark dot in one eye corresponds to a bright dot in the other (Figure 3A). Like V1 complex cells [6, 15, 16], disparity tuning was inverted and attenuated (Figure 3B), causing systematic mispredictions of the stimulus depth (A = 8.83%; CI_95_% = 7.62%, 9.03%).

V1 complex cell attenuation for aRDS is not explained by the canonical energy model, necessitating extensions that have posited additional non-linear stages [16, 17, 18, 19]. However, the BNN naturally exhibited attenuation: by computing the ratio of responses to aRDS versus cRDS, we found striking parallels to V1 neurons [15, 16] (Figure 3C). There was a divergence between the two comparison physiological datasets for low amplitude ratios, with our model closer to Samonds et al. [16]. We speculate that this relates to the disparity selectivity of the sampled neurons: Cumming and Parker [15] recorded closer to the fovea, where sharper disparity tuning functions might be expected. Accordingly, we observed greater attenuation (i.e., lower amplitude ratios) when the BNN was trained on multiway classifications (e.g., seven output units, rather than two), which produced more sharply tuned disparity responses (Figure S3). Together, these results show that inversion and attenuation for anticorrelation appear in a system optimized to process depth in natural images.

The traditional account of aRDS is that they simulate “false matches” that the brain discards to solve the correspondence problem [20, 21]. An alternative possibility, however, is that aRDS responses reflect a computational mechanism for extracting depth. To test this idea, we interrogated the BNN by ordering simple units by their readout weights (Figure 3D) and then visualizing the activity evoked by different stimulus types (Figure 3E). The weighted readout of simple unit activity defines the overall excitatory and suppressive drive to complex units in the network. We found that presenting aRDS led to a striking increase in the activity of the non-preferred simple units, while the activity of the preferred units was more or less unchanged. The consequence of this is that when this activity is read out, it causes increased suppression at the preferred disparity (Figure 3F). This changed the net drive to the complex unit from excitation to suppression (inversion), while the comparatively smaller difference between the excitatory and suppressive drives for aRDS produced a reduced amplitude (attenuation). Thus, attenuation and inversion can be understood based on changing the balance of excitation and suppression, without necessitating additional processing stages.

**Figure 4:**
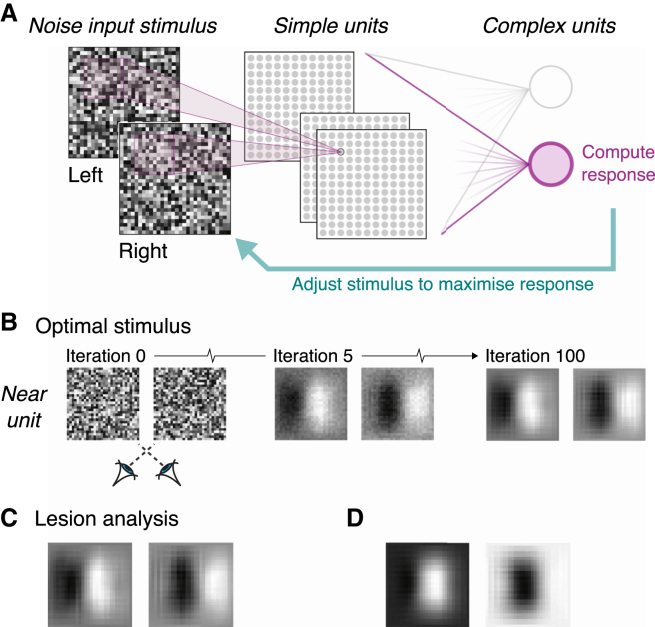
The BNN Is Optimized for the Translation of Image Features that Arises from the Geometry of Binocular Viewing (A) Computing the optimal stimulus for a complex unit. Starting with random noise inputs, the algorithm computed the gradient of complex unit activity with respect to the input images. It iteratively adjusted the inputs to maximize the complex unit’s activity. (B) Snapshots of three iterations during optimization: a consistent on-off pattern emerges in the left and right eyes, horizontally translated to match the preferred disparity of the unit. (C) This pattern remains when “lesioning” the BNN of 25% of the simple units that use position encoding. (D) Removing highly weighted hybrid units leads to input images that are unrealistic.

To ensure that these parallels between the BNN and neurophysiology were not incidental, we tested whether the BNN produces outputs that are well matched to the input stimuli. We used an optimization procedure that started with random noise input images and iteratively adjusted the images such that the activity of a given complex unit was maximized (Figure 4A). Following optimization, the stimuli that best activated the complex units resembled a contrast edge horizontally translated between the eyes (Figure 4B). Thus, the BNN is optimized for the translation of visual features that results from binocular viewing geometry [1]. Importantly, this is achieved using simple units that respond predominantly to different features in the two eyes (Figure 2B), which are traditionally understood as “false” matches (i.e., features that do not correspond to the same physical real-world object). In other words, the BNN extracts depth structure without explicitly “solving the correspondence problem.”

To strengthen this conclusion, we examined the consequences of “lesioning” the BNN by removing 25% of its units. In particular, we removed units with near-zero phase disparities (i.e., the seven units within ±(π/4) of zero phase offset) that are therefore best described as position disparity units that sense similar features in the two eyes. First, we considered decoding performance and found no effect on accuracy (*A*_*Pos*_ = 99.97%, CI_95_% = 99.92%, 100%; p = 0.76; Figure S2D). To situate this null result in the context of arbitrarily removing one-quarter of the units, we also computed decoding performance when we randomly removed seven simple units. In this case, decoding performance dropped considerably (Figure S2D), and there was only 3.8% chance of obtaining a value greater than *A*_*Pos*_. This suggests that the pure position units contribute little to registering the binocular information by the BNN: they are given little weight, so removing them has little effect relative to removing phase or hybrid units. Second, we computed the optimal stimulus for the lesioned BNN (Figure 4C), finding little change relative to the uncompromised network. This null result was not inevitable: removing other simple units resulted in unrealistic images (Figure 4D). Together, these results indicate that the BNN does not critically depend on binocularly matched features.

But how does the BNN extract depth using mismatches, and why should it respond to anticorrelated features? Under the traditional approach, this is a puzzle: a physical object at a given depth would not elicit a bright feature in one eye and a dark feature in the other. However, as we have seen, anticorrelation at the preferred disparity of a complex cell leads to strong suppression. This suggests a role for proscription: by sensing *dissimilar* features, the brain extracts valuable information about unlikely interpretations.

### The BNN Accounts for Unexplained Perceptual Results

**Figure 5:**
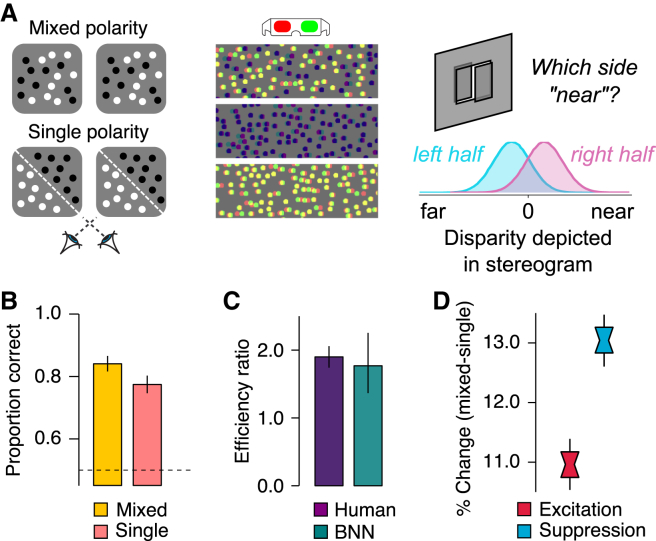
The BNN Mirrors Properties of Human Stereopsis (A) Mixed- versus single-polarity stereograms. Single-polarity stereograms were either all dark or all bright. The task was to discriminate the step arrangement of the stereogram. Anaglyphs were designed for red filter over right eye. (B) Proportion of correct choices of the model after 1,000 trials. (C) Efficiency ratio for mixed versus single stimuli measured psychophysically [22] and for the BNN. (Note: the BNN was optimized on natural images, not on random dot stereograms.) (D) Difference between mixed and single stimuli in terms of the excitatory versus suppressive drive to the non-preferred output unit. Error bars indicate CI_95%_. See also Figure S4.

If proscription has a perceptual correlate, then stereopsis should be affected by the availability of dissimilar features in the scene, an idea we now explore. First, seeing depth should be easier when there is more potential for anticorrelation at the *incorrect* disparity. This logic naturally explains a long-standing puzzle from the psychophysical literature [22, 23] that demonstrated better judgments for stimuli comprising dark and bright dots (mixed polarity) compared to only dark or only bright dots (single polarity) (Figure 5A). This result is difficult to accommodate within the disparity energy model because correlation is largely unaffected by differences in the mean or amplitude of the input signals [23].

We assessed the BNN’s performance on mixed- versus single-polarity stereograms (Figure 5B), finding a benefit for mixed stimuli that was very closely matched to published psychophysical data [22, 23] (Figure 5C). What causes this improvement? As reviewed above, the network depends on the activity of the simple units moderated by readout weights. Presenting mixed- versus single-polarity stimuli increases the simple unit activity, in turn changing the excitatory and suppressive drives to complex units. We found that mixed stimuli produce greater excitation for the preferred output unit and increased suppression to the non-preferred unit (Figure 5D).

We carried out a number of controls to ensure that the BNN’s performance was not artifactual. In particular, contrasting mixed- versus single-polarity stereograms is complicated by low-level stimulus changes (e.g., overall luminance or stimulus intensity range) that could act as covariates that underlie performance [23]. We directly manipulated covariate properties (Figure S4), finding that the benefit for mixed stimuli persisted in all cases. We also tested the specificity of this result to the BNN’s non-linearity [23]. Changing the nonlinearity to an unrectified squaring operation did not change the result (Figure S4). These controls indicate that the improvement for mixed stimuli generalizes over perturbations of the stimuli and network architecture. These results suggest that performance improves for the mixed stimuli because of the opportunity to gain stronger evidence for the true disparity in conjunction with using mismatched features (i.e., dark-to-bright correspondences) as evidence against the incorrect disparity (i.e., proscription). This could be implemented in vivo using suppressive inputs to V1 neurons [24].

**Figure 6:**
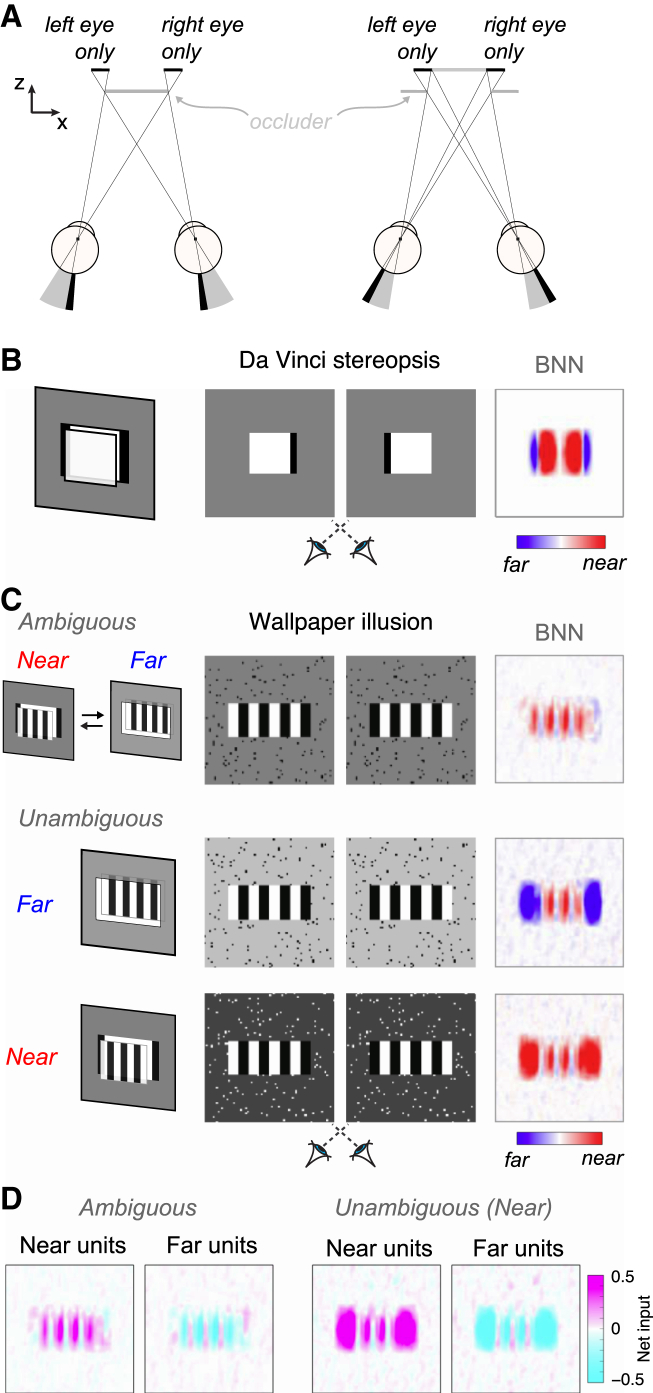
The BNN Can Predict Depth Order When Disparity Is Ill Defined or Ambiguous (A) Illustration of occlusion around the edges of objects. (B) da Vinci stereopsis. Left: illustration of half-occlusions (black flanks) produced by viewing geometry; center: da Vinci stereograms for cross-eyed fusion; right: depth map from the BNN. (C) Wallpaper illusion. Top: ambiguous pattern. The vertical stripes can be matched by a nasal or temporal shift, making both near and far global matches valid. Cross-eyed fusion allows the reader to experience alternation. The BNN does not detect a clear depth. Bottom: biasing perception by changing background luminance leads to a concomitant shift in the BNN’s interpretation. (D) The net drive between excitation and suppression that underlies the shift in prediction, contrasting the ambiguous case and disambiguated case. Note: for all of these examples, it is clear that the BNN has not “reproduced” the percept; rather, the network provides key signals that may provide the foundations for typical percepts. See also Figure S5.

A second line of evidence in favor of proscription comes from considering situations regarded as too difficult for accounts of stereopsis based on peak correlation. Under natural viewing, certain features are visible to one eye but not the other (Figure 6A). The brain exploits such unpaired elements, “da Vinci stereopsis,” to support depth perception [25, 26]. However, these stimuli pose a severe challenge to traditional stereo algorithms because there are no matching features [27]. We tested the BNN on a stimulus with unpaired features around a zero-disparity target (Figure 6B). Because the target was not displaced in depth, there are no binocular corresponding features to compute the depth relationship. However, the BNN predicted the ordinal depth structure experienced by observers for the edge regions (Figure 6B), and this result generalized to stimuli with different luminance configurations (Figure S5). The BNN thus extracts critical signals that may provide the foundation for a full perceptual interpretation when used in conjunction with processes such as figure-ground segmentation at further stages of visual processing [28, 29].

Finally, we tested the BNN on the classic “wallpaper illusion” [30], in which periodic patterns yield ambiguous depth percepts. When disparity matches were ambiguous, the disparity-sign map did not identify a clear depth edge (Figure 6C). However, by manipulating the background luminance to bias matching [27], we found that the BNN predicted the perceptual interpretation of the stereograms in the edge regions. This was achieved by changing the net excitatory-suppressive drive at the half-occluded regions, where disambiguation occurs (Figure 6D). This is compatible with early processing of half-occluded edge regions in V1, providing an initial basis for subsequent depth interpolation supported by extrastriate cortex [31] or via recurrent connectivity within V1.

Together, these results indicate that, without being trained on such displays, the BNN’s combination of detection and proscription provides a natural foundation for typical percepts. The simple units of the BNN exploit receptive fields that capture a continuum of similarities and differences between the binocular images, contrasting with the standard approach to binocular vision that emphasized the importance of correct matches. Although individual units in the BNN are not specialized to identify the same feature in the two images, the aggregate readout activity classifies depth with high accuracy, and complex units respond best to physically realistic displacements of a single object.

### Detection and Proscription Combine to Facilitate Sensory Estimation

We have seen that the BNN generalizes well from its training set and accounts for both neurophysiological and perceptual phenomena. However, the network’s multiple parameters may act as a barrier to a detailed understanding of its operation. We therefore sought to explain the BNN’s behavior in theoretical terms by deriving a low-parameter closed-form model that captures its key characteristics. Our starting point was to observe that a low-dimensional rule relates the BNN’s simple units and their readout: weights are proportional to the cross-correlogram between the (left and right) receptive fields (*R* = 0.89; p < 0.001) (Figure S6).

The key intuition behind this relationship is that receptive fields capturing a positive correlation at disparity $\delta_{i}$ (i.e., the lag of the cross-correlogram) should be read out by a complex unit with preferred disparity $\delta_{i}$ using a positive (i.e., excitatory) weight. Conversely, if the simple unit captures a negative correlation at disparity $\delta_{i}$, the complex unit should read out its activity using a negative (suppressive) weight. In other words, the same simple units can be read out with detection or proscription to provide a population-based estimate of the depth of the viewed scene.

We show formally (see STAR Methods) that using weights determined by the cross-correlogram of the left and right receptive fields is optimal under reasonable assumptions and propose a binocular likelihood model (BLM) captured by a simple equation,

$$\text{log}L\left(\delta \right)=\sum_{i=1}^{N}r_{i}{\left(W_{L}⋆W_{R}\right)}_{i}\left[\delta \right].$$

**Figure 7:**
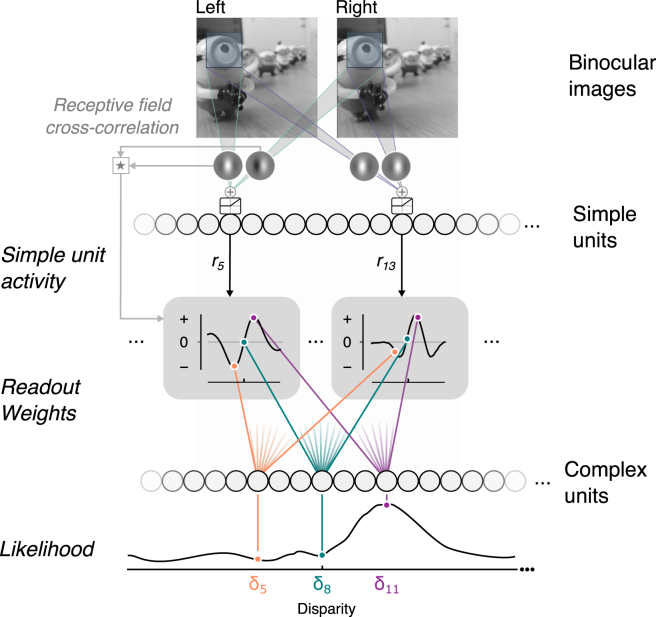
Binocular Likelihood Model Input images are processed by a population of simple units that perform linear filtering followed by nonlinear rectification. The activity of a given simple unit (*r*_*i*_) is read out by multiple complex units. A simple unit’s readout weights vary over complex units, where the readout weight is defined by the cross-correlation of the simple unit’s left and right receptive fields. The activity of the population of complex cells encodes the likelihood function for stimulus disparity. See also Figure S6 and Figure S7.

This relationship states that the activity of a complex unit that prefers a given disparity δ (expressed as a log likelihood, L(δ)) is given by a weighted sum of simple unit activity, $r_{i}$. The weights correspond to the cross-correlation, ${\left(W_{L}⋆W_{R}\right)}_{i}$, between the left and right receptive fields of simple unit *i* at disparity δ (Figure 7). To demonstrate the model, we implemented an instantiation that produces disparity tuning curves for correlated and anticorrelated RDS that closely resemble V1 complex cells (Figure S7). This instantiation included a single spatial frequency channel, so the model does not require pooling across spatial scales to exhibit attenuation for aRDS. The model’s key parameters are simply the receptive fields of the input units. This suggests that a fixed, stimulus-independent architecture explains key binocular phenomena, possibly without supervised learning.

## Discussion

Traditional understanding of stereopsis at the computational, neural, and perceptual levels has focused on the idea that peak correlation should be used to identify similar features and discard false matches. The logic underlying this approach is based on inverting the geometry that maps objects at different locations in space onto different portions of the two retinae. However, here we show that envisaging neurons as units that match up the features of objects in the world fails to account for known properties of neurons and overemphasizes the role of similarity in a system whose fundamental benefit lies in differences between the images sensed by the two eyes.

We demonstrate that V1 neurons have properties ideally suited to extract binocular information, rather than simply searching for matching features. We formalize a binocular likelihood model that provides a unifying account for previously puzzling properties of V1 neurons as well as perceptual phenomena that challenge the standard approach. This model highlights the interplay between feature detection and proscription for perceptual inference. This mix of evidence for and against likely interpretations may represent a general strategy for perceptual integration both within and between sensory modalities.

### Understanding the Functional Role of Sensory Neurons

Understanding the coding strategies of sensory neurons represents a long-standing challenge. A historically pervasive idea is that sensory neurons act as “feature detectors,” signaling evidence for the occurrence of a particular feature in the environment [32, 33]. For instance, orientation-selective neurons could indicate the presence of a particular tilted edge in a visual display [34]. It has long been recognized that natural images shape this selectivity [35, 36], with neural responses optimized for efficient representation of the statistical regularities of the environment [37, 38].

Here we take the approach of quantifying the information conveyed by early sensory neurons that are sensitive to binocular disparity using information analysis, and then implementing a neural network optimized by exposure to natural images. This provides insight into the functional purposes of disparity representations at the neural and perceptual levels. Our findings on the utility of hybrid receptive fields for disparity encoding are consistent with work that used dimensionality reduction to estimate the optimal disparity filters [39]. In particular, our observation that hybrid units capture greater Shannon information is consistent with the idea that hybrid encoding maximizes disparity estimation accuracy. Moreover, hybrid receptive fields are suggested to minimize the statistical redundancy of binocular responses [40, 41, 42], suggesting an additional factor driving the brain’s use of hybrid units.

### Understanding the Encoding Properties of the BNN

Previously it was suggested that phase encoding is used to sense “impossible” stimuli. In particular, Read and Cumming [12] made an important proposal that key depth information is conveyed by positional disparities, with phase disparity used to select between alternative positional signals in cases of ambiguity. They suggested that this would filter out “false” matches and thereby solve the correspondence problem. In contrast, our model is based on the combination of feature detection and proscription, rather than using mismatches as a veto. As we have shown, extracting depth structure can be achieved without units that register pure positional disparities: only 3 of 28 simple units responded to position offsets without phase offsets (Figure 2B), and removing units with small phase offsets had little consequence on the performance of the network (Figure 4C).

More generally, it is important to ask why the BNN, optimized by natural images, uses hybrid encoding for its simple units. The traditional exposition of binocular vision starts from the convenient geometry of how a small number of isolated points in the world project into the retinal images sensed by the two eyes. Models of binocular vision are typically built upon the logic of inverting this mapping based on establishing the “correct” matches. However, the BNN suggests that the diet of early visual neurons consists almost entirely of mismatched features: the one “true” set of correspondences between the two eyes is engulfed by a preponderance of mismatches.

When interpreting the properties of the BNN, it is important to recall that the network learned the relationship between specific inputs (i.e., one natural image set) and the optimization objective (i.e., a particular discrimination task). Systematically changing either would change the learned model. Nevertheless, the BNN generalized to a different stimulus set (random dot patterns) and had properties mirroring neurophysiology. It is interesting that the BNN’s receptive fields are vertically oriented. Although this makes sense when capturing horizontal disparities, real V1 binocular neurons have varied orientation tuning preferences [9]. This difference may relate to the fact that the BNN is constrained to optimize one task (disparity discrimination) while V1 neurons are required to support many. It will be interesting to test how defining models for multiple objectives (e.g., estimating the orientation of features tilted in depth) affects encoding properties. For instance, future work might test whether units become specialized for particular functions versus developing joint-encoding characteristics. This might most straightforwardly be applied to proscriptive processing for motion estimation (given the strong computational similarities between disparity and motion [43]) but may also extend to other feature dimensions.

### Relation to the Disparity Energy Model

The disparity energy model [6, 7, 8] has long provided the foundation for understanding binocular vision. Although modifications have been proposed to accommodate a number of electrophysiological observations [16, 18, 19], the basic architecture has remained unchanged. Moreover, the link between the implementation and the computational goal of estimating depth has been left obscure.

Here we developed an approach that exploits the same computational building blocks as the traditional model (i.e., linear filters for binocular summation followed by rectification). However, the BLM uses a weighted readout scheme, in which activity can be combined via excitatory or suppressive weights onto a population of complex cells. The main deviations from the traditional model are (1) the existence of multiple simple cell-like neurons, as opposed to the quadrature pairs originally proposed, (2) the incorporation of variable weights that can be suppressive, and (3) the complex unit’s use of responses from simple units that do not have the same preferred disparity (because simple units convey information about multiple disparities). These characteristics are not part of the classical energy model but strongly align with modifications suggested in light of neurophysiological evidence [18, 24, 44, 45, 46]. As we have shown, by using a model optimized to estimate depth, readout weights can be derived directly from the model’s encoding properties. The fact that doing this reproduces properties of simple and complex cells measured in vivo suggests that the visual system has been optimized by similar constraints.

The role we demonstrate for proscription is consistent with evidence that binocular V1 neurons are modulated by excitatory and suppressive components [24]. That suppression lags behind excitation by ∼7 ms [45] suggests that it is initiated at very early stages of processing. In particular, the proscriptive registration of dissimilarities could drive suppression of unlikely depths via inhibitory interneurons. The necessity of an additional synapse (via interneurons) would impose a small temporal delay, but this delay is less than would be expected for extrastriate feedback. The BLM suggests that the properties of suppressive inputs shape the inversion and attenuation of complex cell tuning curves for aRDS. Where suppressive input is strong, we expect a clear inversion of the tuning curve but little attenuation. Conversely, where suppressive input is weak, such that excitation and suppression are nearly balanced, the tuning curve would be severely attenuated. In this case, the close balance between excitatory and suppressive inputs means that highly attenuated cells take longer to cross their firing threshold. This is consistent with evidence from barn owls that longer onset latencies are associated with high attenuation [47].

Finally, the BLM predicts that anticorrelation masks the registration of a correlated disparity signal. Previous work pitted cRDS against aRDS to produce zero net correlation in the display. Participants can judge depth in such displays, leading to the suggestion of an additional mechanism separate from correlation [48]. In contrast, the BLM posits a single mechanism and exploits anticorrelation to facilitate the interpretation of depth. We predict that the masking effects of anticorrelation are tuned (i.e., that anticorrelated disparities are more suppressed than others) and that spatial limits on masking from anticorrelation are set by V1 complex cell receptive fields.

### Relation to Binocular Rivalry

Our mechanistic account of the early stages of binocular vision suggests a natural link to work on binocular rivalry. Traditionally, the study of rivalry and stereopsis have been separate [49, 50], although recent work has suggested computational links between them [51]. Here we show that proscription is likely to be a key constituent of normal disparity processing. This suggests that stereopsis and rivalry sit along a spectrum of binocular responses mediated by inhibition. This is compatible with work on the perception of visual appearance [52] and suggests a link to GABA-mediated inhibition related to binocular rivalry. For instance, there is a strong association between human V1 GABA concentration (quantified by magnetic resonance spectroscopy) and monocular percept duration [53]. Furthermore, temporary monocular deprivation leads to reduced V1 GABA [54]. Therefore, it seems plausible that inhibitory mechanisms in V1 are related to processing binocular incongruence. It will be interesting to test how the mechanisms that we propose are implemented physiologically, and whether these support a unifying axis between rivalry and stereopsis.

### Relation to Cue Integration and Multisensory Processing

Finally, it is worth noting that neuronal tuning to properties that appear inconsistent with the physical structure of the world are not limited to binocular disparity. In particular, neurons can be tuned to the same or opposite features for different visual cues and/or between sensory modalities [55, 56, 57]. For instance, certain neurons in macaque area MSTd respond maximally to the same direction of motion when specified either by visual or by vestibular cues (“congruent”), while others (“incongruent”) have opposite direction preferences between modalities [57]. As with the discussion of phase disparity, “incongruent” neurons are puzzling because they respond best to stimulation that could not be caused by a single physical object.

The inference framework that we provide for binocular vision suggests an important role for neurons that encode proscriptive features. We hypothesize that a similar mechanism is used when combining different cues (e.g., disparity and texture) or sensory modalities (e.g., vision and touch). Specifically, neurons form a continuum of responses (ranging from “congruent” to “incongruent”) analogous to “hybrid” disparity encoding. These encoding neurons can be read out by a population of units that integrate signals from different cues. This can broadly be conceptualized as a type of causal inference based on explaining away [58] and links to suggestions about providing a mechanism for discounting irrelevant properties of viewed stimuli [59].

### Conclusions

Early sensory neurons are broadly understood as optimized to capture the physical properties of the surrounding environment. Within this context, neural tuning to elements that do not relate to physical objects represents a significant puzzle. Using an optimal information framework, we demonstrate the importance of proscription: neural responses that provide evidence against interpretations incompatible with the physical causes of sensations. We demonstrate the role of these “what not” responses in a neural network optimized to extract depth in natural images. We show that combining detection with proscription provides a unified account of key physiological and perceptual observations in 3D vision that are unexplained by traditional approaches. We capture the encoding and readout mechanisms in simple analytical form and propose that marrying detection with proscription provides an effective coding strategy for sensory estimation.

## STAR★Methods

### Key Resources Table

**Table:** 

REAGENT or RESOURCE	SOURCE	IDENTIFIER
**Software and Algorithms**		

Theano Python library	[60]	http://deeplearning.net/software/theano/
Routines for optimizing the binocular neural network	This paper	https://doi.org/10.17863/CAM.8538
Implementation of the binocular likelihood model	This paper	https://doi.org/10.17863/CAM.8538

### Contact for Reagent and Resource Sharing

Further information and requests for resources and reagents should be directed to and will be fulfilled by the Lead Contact, Andrew E. Welchman (aew69@cam.ac.uk).

### Method Details

#### Information Theoretic Analysis

##### Individual Simple Units

We sought to formalize the idea that information encoded in the responses of binocular simple units is not restricted to the preferred disparity. To do so, we computed the Shannon information *I* between broadband stimuli *s* with varying disparity δ and simple unit responses *R*,

(1) $$I\left(R,s_{\delta }\right)=\sum_{i}p\left(r_{i}|s_{\delta }\right)\text{log}\frac{p\left(r_{i}|s_{\delta }\right)}{p\left(r_{i}\right)},$$

where $r_{i}$ denotes the firing rate of the simple unit. The resulting information indicates how well a particular disparity is encoded in the response of the simple unit. In this demonstration, the receptive fields were parameterized as two-dimensional (x,y), vertically oriented Gabor functions,

(2) $$W\left(x,y\right)=e^{\left({\left(x-x_{0}\right)}^{2}+y^{2}\right)/2\sigma^{2}}\text{cos}\left(2\pi f\left(x-x_{0}\right)+\phi \right),$$

where σ denotes the Gaussian envelope width, $x_{0}$ denotes the position, *f* the spatial frequency, and ϕ denotes the phase of the receptive field. To define the disparity encoded by the simple unit, we varied the phase and/or position, and kept the remaining parameters constant. Varying the position parameter introduces a simple translation in the receptive field, while varying the phase causes a change in the internal structure of the receptive field.

We computed the information carried by a simple unit with preferred disparity of 4 pixels defined by a either a position shift or a phase shift. For this simulation, the receptive field envelope, σ, was set to 5 pixels and the frequency, *f*, was set to 0.05 cycles/pixel. The stimulus set consisted of 100,000 uniform random dot images with disparities between − 20 and 20 pixels. For both encoding mechanisms, we observed that individual simple units convey information about non-preferred disparities (Figure 1C). This highlights that the activity of simple units selective for a particular disparity could contribute to the activity of complex units tuned to different disparities.

##### Population of Simple Units

In the previous section we examined information at the single unit level. Next, we demonstrate how much information is encoded across a small population of simple units (N=5) with position, phase, and hybrid disparity encoding. We used a small number of units for computational convenience, as the amount of memory required to store the full stimulus-response distribution increased exponentially with the number of units (simulating a population of 10 units, for instance, would require a prohibitive 80 gigabytes of RAM memory). An alternative to study information in larger neural populations would be to use other measures such as the linear Fisher Information – a quantity that is inversely related to discrimination thresholds, and that can be efficiently computed if responses follow a distribution of the exponential family with linear sufficient statistics (e.g., [61]). However, we chose to use Shannon Information to avoid focusing on discrimination tasks and obviate further assumptions about the response distribution.

Although we are now working at the level of multiple simple units, Equation 1 can still be used – the difference is that the response is a vector of activities of multiple simple units, so the underlying probability distributions are multidimensional. Because we are not interested in the information about individual stimulus disparities, but rather how well all disparities are encoded, we integrate over the stimulus disparity,

(3) $$I\left(R,S\right)=\sum_{\delta }\sum_{i}p\left(r_{i}|s_{\delta }\right)\text{log}\frac{p\left(r_{i}|s_{\delta }\right)}{p\left(r_{i}\right)}.$$

We generated populations of simple units with (i) position shifts, (ii) phase shifts, or (iii) a combination of both (hybrid encoding). The Gaussian envelope width, σ, and the spatial frequency, *f*, were kept constant, and only the position $x_{0}$ and the phase ϕ parameters were allowed to vary.

We examined information encoded under two schemes. First, we computed the information under the assumption of uniformly spaced simple units. This ensures minimal overlap between the tuning curves of the simple units, and therefore avoids redundancy (i.e., the suboptimal case where two or more units in the population have very similar tuning curves). Next, we examined information without imposing this uniform spacing, and allowed the simple units to assume random tuning profiles. We did this by generating 1,000 populations for which the position and/or phase shifts (according to the encoding mechanisms under evaluation) were randomly drawn from a uniform distribution. This yielded a distribution of information values for each of the mechanisms. As expected, we observed higher information values for the uniformly distributed population (Figure 1D, horizontal lines) when compared to random populations (Figure 1D, bar graph). In both cases, we found that hybrid populations carried the most information about the disparity imposed in our stimulus set (Figure 1D).

#### Naturalistic Binocular Images

We generated naturalistic stereoscopic images using 100 light-field photographs extracted from the Light Field Saliency Database [14] (http://www.eecis.udel.edu/∼nianyi/LFSD.htm). The dataset comprised images of a variety of indoor and outdoor scenes—representative stereo pairs are provided in Figure S1—and the corresponding depth maps. First, each RGB image (1080-by-1080 pixels) was converted to gray-scale values and down-sampled at the resolution of the corresponding depth map (328-by-328 pixels). Thereafter, we used the information provided by the depth map to render stereo pairs with arbitrary disparity range. From each light-field acquisition, we extracted a series of images focused at different points in depth, and rendered stereoscopic pairs by shifting the pixels of the original image by an amount proportional to the value of the depth map, restricting the maximum shift to 10 pixels. Pixels that were revealed behind occluded regions (by displacing image features in depth) were filled using linear interpolation. To prevent interpolation from affecting the training procedure, we excluded image patches for which more than 5% of the pixels were interpolated.

This method produced 200 stereo pairs. From these images we extracted 38,000 different pairs of smaller image patches (30-by-30 pixels). To ensure accurate disparity information, we excluded image patches with low variance of pixel intensity (gray level s.d. threshold = 20). All image patches were then scaled so that pixel intensity values were contained in the interval between –1 and 1, and randomly divided into training and test sets, as described below.

We did not use standard two frame stereo datasets (e.g., Middlebury datasets) given that these contain a large range of disparities, making it difficult to obtain sufficiently large training sets for a given set of disparity values. We restricted the network to work on a small number of individual disparities for which we could provide training data. Rendering stereo pairs from the corresponding depth map, as described above, allowed us to generate images with arbitrary disparity range, and therefore increase the number of class exemplars available to train the network. Additionally, native two frame stereo datasets are typically composed of a comparatively small number of photographs, which could lead to exploring a narrow portion of the space of natural image statistics. This would affect the properties of the network and the degree to which it could generalize to other stimuli.

#### Binocular Neural Network

##### Architecture

The binocular network was implemented using Theano [60], a library for efficient optimization and evaluation of mathematical expressions. We used a simple convolutional neural network that comprised (i) an input layer, (ii) a convolutional-pooling layer and (iii) an output logistic regression layer (Figure 2A). The input is convolved with a series of kernels to produce one output map per kernel (which we refer to as convolutional maps). The use of convolution means that each kernel is applied at all different locations of the input space. This significantly reduces the number of parameters that need to be learned (i.e., we do not parametrize all possible pairwise connections between layers) and allows the network to extract a given image feature at all different positions of the image.

Inputs were image patches (30x30x2 pixels; the last dimension carrying the left and right images) extracted from stereoscopic images. In the convolutional layer, binocular inputs are passed through 28 binocular kernels (19x19x2 pixels) producing 28 output maps (12x12 pixels). This resulted in 4,032 units (28 maps of dimensions 12x12 pixels) forming 2,911,104 connections to the input layer (4,032x19x19x2 pixels). Since this mapping is convolutional, this required that 20,244 parameters were learned for this layer (28 filters of dimensions 19x19x2 plus 28 bias terms). We chose units with rectified linear activation functions since a rectifying non-linearity is biologically plausible and necessary to model neurophysiological data [62]. The activity, *a*, of unit *j* in the $k^{th}$ convolutional map was given by:

(4) $$a_{j}^{\left(k\right)}={\left(w^{\left(k\right)}s_{j}+b_{j}^{\left(k\right)}\right)}_{+}$$

where $w^{\left(k\right)}$ is the 19x19x2 dimensional binocular kernel of the $k^{th}$ convolutional map, $s_{j}$ is the 19x19x2 binocular image captured by the $j^{th}$ unit, $b_{j}$ is a bias term and ${\left(.\right)}_{+}$ denotes a linear rectification non-linearity (ReLU). Parameterizing the left and right images separately, the activity $a_{j}\left(k\right)$ can be alternatively written as:

(5) $$a_{j}^{\left(k\right)}={\left(w^{\left(Lk\right)}s_{j}^{L}+w^{\left(Rk\right)}s_{j}^{R}+b_{j}^{\left(k\right)}\right)}_{+}$$

where $w^{\left(Lk\right)}$ and $w^{\left(Rk\right)}$ represent the $k^{th}$ kernels applied to left and right images (i.e., left and right receptive fields), while $s_{L}^{j}$ and $s_{R}^{j}$ represent the left and right input images captured by the receptive field of unit *j*.

The convolutional layer was followed by a max-pooling layer that down-sampled each kernel map by a factor of two, producing 28 maps of dimensions 6-by-6 pixels. Finally, a logistic regression layer (1,008 connections; 36 per feature map, resulting in 1,010 parameters including the bias terms) mapped the activities in the pooling layer to two output decision units. The vector of output activities *r* was obtained by mapping the vector of activities in the pooling layer a via the weight matrix *W* and adding the bias terms *b*, followed by a softmax operation:

(6) r=softmax(Wa+b)

The predicted class was determined as the unit with highest activity. For *N*-way classification, the architecture was identical except for the number of output units of the BNN.

##### Training Procedure

The input stereo pairs were first randomly divided into training- (70%, 26,600 pairs), validation- (15%, 5,700 pairs) and test- (15%, 5,700 pairs) sets. No patches were simultaneously present in the training, validation, and test sets. To optimize the BNN, only the training and validation sets were used. We initialized the weights of the convolutional layer as Gabor filters with no differences between the left and right images. Therefore, initialization provided no disparity selectivity. With *x* and *y* indexing the coordinates in pixels with respect to the center of each kernel, the left and right monocular kernels $W^{L}$ and $W^{R}$ of the $j^{th}$ unit were initialized as

(7) $$w_{j}^{L}=w_{j}^{R}=e^{-\left(x^{\text{′}2}+y^{\text{′}2}\right)/\left(2\sigma^{2}\right)}\text{cos}\left(2\pi fx^{\prime }+\phi \right)$$

with *f* = 0.1 cycles/pixel, σ = 3 pixel, θ = π/2 radians, $x^{\prime }=x\text{cos}\left(\theta \right)+y\text{sin}\left(\theta \right)$, $y^{\prime }=-x\text{sin}\left(\theta \right)+y\text{cos}\left(\theta \right)$, and ϕ the phase of the cosine term of each unit, which was equally spaced between 0 and π. The bias terms of these units were initialized to zero. During training we did not constrain the filters to any particular morphology, neither did we constrain properties such as spatial frequency selectivity. In the logistic regression layer, the weights and bias terms were all initialized to zero.

The BNN was trained using mini-batch gradient descent with each batch comprising 100 examples (50 examples of each class). For each batch, we computed the derivative of the loss function with respect to parameters of the network via back-propagation, and adjusted the parameters for the next iteration according to the update rule

(8) $$w_{i+1}=w_{i}-\alpha \langle \frac{\partial L}{\partial w_{\left(D_{i}\right)}}\rangle$$

where α is the learning rate, and $\langle \partial L/\partial w_{\left(D_{i}\right)}\rangle$ is the average over the batch $D_{i}$ of the derivative of the loss function with respect to the *w*, evaluated at $w_{i}$. The learning rate α was constant and equal to 0.001.

After evaluating all the batches once—completing one epoch—we tested the BNN using the validation image dataset. We repeated this process for a maximum of 1,000 epochs. Initially, the maximum number of iterations allowed without improvement was set to 10,000. To allow exhaustive optimization, this limit was increased by a factor of 2 every time there was an improvement of 0.5% in performance as tested in the validation set.

##### Evaluation

We tested the BNN using both natural and synthetic images. For natural images, we tested it using 5,700 held-out patches on the test image dataset (i.e., these exemplars were not used for training or validating the network). For comparison with neurophysiological observations, we also tested the BNN using random-dot stereogram patches. This test set consisted of 6,000 randomly generated stereograms containing a mixture of dark and bright dots on a gray background (dot size = 1 pixel; dot density = 50%).

For comparison with psychophysical observations, we also tested the BNN with large random-dot stereograms depicting a step-edge (240-by-240 pixels). The dot size was set to 8 pixels and the dot density was approximately 15%. No occlusion between the dots was allowed. The step disparity was set to 2 pixels. Disparity noise sampled from a Gaussian distribution (s.d. = 8 pixels) was added to increase task difficulty. Stereograms could contain bright dots, dark dots (single polarity cases) or an even mixture of both (mixed polarity case) on a uniform mid-gray background. Bright, dark, and mid-gray pixels corresponded to values of + 1, − 1 and 0, respectively. Differences in the response to mixed- and single-polarity stereograms could be affected by differences in mean luminance or contrast. We sought to rule out such effects by performing control analyses where these properties were matched. In particular, we report the results obtained when the mean luminance (DC) was removed, as differences in DC can have a drastic effect on the population responses [23]. Similar results were obtained when single-polarity stereograms were scaled to have the same peak-to-trough values (i.e., pixel intensities varied from − 1 to + 1, producing a range of 2), and scaled to match the range of the mixed polarity stereograms after we had removed the mean luminance. Figure S4 compares results obtained with different manipulations of the images.

#### Modeling Binocular Receptive Fields

The receptive fields of simple units in the BNN were not constrained to develop a particular structure (i.e., Gabor functions) during optimization – they could in principle develop any kind of morphology. We therefore assessed whether the receptive field structure mirrored that found in simple cells in primary visual cortex. In particular, we set out to test (i) if the receptive fields were well approximated by Gabor functions, and (ii) what kind of encoding mechanism they develop – i.e., position, phase or hybrid encoding.

We started by assessing whether the receptive fields were well approximated by Gabor functions. To reduce the number of free-parameters, we examined the horizontal cross-section of the receptive field, and fit a 1-dimensional Gabor function,

(9) $$W=A\times e^{-{\left(x-x_{0}\right)}^{2}/\left(2\sigma^{2}\right)}\text{cos}\left(2\pi f\left(x-x_{0}\right)+\phi \right).$$

We used a two-stage procedure for optimization. First, we ran a coarse grid-search to find a good initial guess for the parameters, whereby the combination of parameters with lowest sum of squared errors was selected. Then, taking the grid-search estimates as initial guesses, we estimated the final parameters using bound constrained minimization. The constrained parameters were the amplitude (0<A<+∞), the center of the envelope $\left(min\left(x\right)<x_{0}<max\left(x\right)\right)$, the phase (−π<ϕ<π) and the frequency, which was constrained to an interval of ±10% around the peak of the Fourier transform of the receptive field profile. To assess whether disparity was encoded via position and/or phase shifts (Figure 1B), we subtracted the position/phase parameters between the left and right receptive fields. The phase parameter was wrapped to [−π,π].

To address consistency with neurophysiology, we examined the spatial frequency bandwidth of the receptive fields learned by our model. We quantified spatial frequency bandwidth using two methods. First, we used a non-parametric approach of computing the spatial frequency tuning curve for each filter, and then determining the corresponding bandwidth (FWHM). We found that the spatial frequency bandwidth values were plausible when compared to the bandwidth of V1 neurons [63] (average bandwidth = 2.32 octaves; values ranged from 1.58 to 3.44 octaves). As a confirmatory procedure, we used a parametric approach based on the standard deviation and the frequency parameters of the Gabor fits. This yielded near-identical results, although 13/56 filters could not be evaluated using this method as they produced NaN estimates.

#### Varying the Number of Simple Units and Testing the Importance of Positional Disparities

When defining the architecture of the BNN, we arbitrarily set the number of simple unit types to 28. To ensure that our results hold in a more generalized manner, we additionally trained similar versions of the Binocular Neural Network while varying the number of simple unit types. The remaining parameters of the network were kept constant. After optimization, we found a similar pattern of results: we achieved high classification accuracies (Figure S2A), and the binocular receptive fields developed a combination of phase and position disparities (Figures S2B and S2C).

Relating simple unit properties (i.e., their receptive fields) to the readout of their activity is a key step in understanding the computation performed by the network. We chose to deploy the network with 28 types of simple units as opposed to the models with fewer units. This was because it provided a richer substrate to determine the relationship between simple units properties and their readout, and allowed us to perform a ‘lesion’ analysis of the network where performance was not uniquely dependent on a very small number of units. With fewer units (e.g., 8), performance when dropping units would have become unstable.

#### Estimating Correlated versus Anticorrelated Amplitude Ratios

Complex units in the BNN responded more vigorously to correlated (cRDS) than anticorrelated stereograms (aRDS) (Figure 3A), a phenomenon that is observed in disparity selective V1 complex cells [15, 16]. We examined whether the degree of attenuation observed in our network was compatible with electrophysiological data. Attenuation is commonly assessed by modeling tuning curves for aRDS and cRDS, and then evaluating the ratio between the corresponding amplitudes [15, 20, 47]. Therefore, we modeled the tuning curves using Gabor functions (similar to those used to model the binocular receptive fields) and computed the ratio between the amplitude parameter for correlated and anticorrelated stimuli. We started by generating disparity tuning curves for each complex unit by computing the activity elicited by correlated or anticorrelated random-dot stereograms (50% dot density) with disparities ranging from − 20 to 20 pixels (100 trials per disparity) (Figure 3B). To avoid relying on a single fit per complex unit, we used bootstrapping to generate 5,000 resampled tuning curves, and we fit a Gabor to each sample. The average explained variance of the fits to the disparity tuning curves was $R^{2}=0.945$ ($R^{2}=0.93$ for cRDS and $R^{2}=0.96$ for aRDS). Based on these parameters, we computed the respective amplitude ratios by dividing the amplitudes for aRDS by the amplitudes for cRDS. We finally arrived at a distribution of amplitude ratios (Figure 3C) by pooling the data across complex units.

#### N-Way Classification

In addition to the binary case, we also trained a network to perform *N*-way classification. The only change required to the network was an increase in the number of output complex units. In particular, we optimized a network for 7- and 11-way classification. In these cases, the complex units of the network also display inversion and attenuation for anticorrelated random-dot stereograms, with comparable but more variable amplitude ratios (Figure S3). We found that the corresponding tuning curves featured abrupt changes in selectivity, and some were not well described by Gabor-like profiles. We note that this is also the case in cortex (i.e., that Gabor functions do not always describe disparity tuning well). However, the abrupt variations in tuning could be alleviated by varying the temperature of the *softmax* nonlinearity, or by defining the *N*-way classification problem to operate over a broader disparity space.

#### Computing Optimal Stimuli

To confirm that the model was well tuned to extract physical binocular disparities, we computed input images that could best activate the complex units of our model. The intuition is that we can visualize what inputs are most efficient in driving a given complex unit, and thereafter evaluate whether the input is sensible. The objective function is therefore the activity of a given complex unit, which we want to maximize. Equivalently, for an output unit *j*, we minimized the negative of its input:

(10) $$L_{j}=-\left(W_{j}a+b_{j}\right)$$

where *a* is the vector of simple unit activities, $W_{j}$ is the readout weight matrix for the $j^{th}$ complex unit, and $b_{j}$ is the bias term. The goal is thus to find an input image that minimizes $L_{j}$ (i.e., maximizes the complex unit activity; Figure 4A). We did this via gradient descent: we started with a random noise input image, *x*, computed the gradient of the loss function with respect to the input image, and adjusted the latter according to the update rule:

(11) $$x_{i+1}=x_{i}-\alpha \frac{\partial L}{\partial x}$$

where α is the step size (empirically set to 1). We limited the number of iterations to 100 as this was enough to ensure that optimization reached a stable image configuration (i.e., the correlation between the stimulus in two consecutive iterations saturated at 1).

The stimuli that best activated the complex units resembled contrast edges horizontally translated between the eyes, in the direction consistent with the preferred disparity of the complex unit (Figure 4B). This is consistent with detecting positional offsets. The structure of the optimal stimuli was very similar across the eyes, indicating that stimuli with non-physical (i.e., phase) disparities are not ideal to activate the BNN’s complex units.

#### Step-Edge Depth Discrimination and Depth-Sign Maps

In its original form, the BNN takes a 30-by-30 input image patch and produces a binary output corresponding to the predicted disparity (*near* or *far*). Once trained, however, convolutional neural networks can be applied to higher dimensional inputs, without requiring any changes in the parameters of convolutional layers. We took advantage of this convenience to test the BNN with larger binocular inputs. The only required modification to the BNN happened in the readout layer, where we applied the mean readout weight for each simple unit in an element-wise manner. This resulted in two output activity maps – one for near disparities (*near* map), and another one for far disparities (*far* map). More formally, the vector of activities in the $j^{th}$ output map was defined as:

(12) $$a_{out}^{\left(j\right)}=\sum_{\left(k=1\right)}^{28}a_{conv}^{\left(k\right)}{\hat{w}}_{out}^{\left(kj\right)}+b^{\left(j\right)}$$

where $a_{conv}^{\left(k\right)}$ is the vector of activities in the $k^{th}$ convolutional map, ${\hat{w}}_{out}^{\left(kj\right)}$ is the mean readout weight between the $k^{th}$ convolutional map and the $j^{th}$ output unit, and $b^{\left(j\right)}$ is the vector of bias terms of the $j^{th}$ output unit. Finally, we combined the two output maps by element-wise subtracting the activities of the *near* map from the *far* map, so that positive values reflect higher *near* activity, while negative values reflect higher *far* activity.

#### Relationship between Simple Unit Selectivity and Readout

The activity of complex units in the network depends on the readout of the activity of the population of simple units. We assessed whether there was a relationship between the receptive fields of simple units and the corresponding readout weights. Take, for instance, the complex unit that responded to *near* stimuli: how does this complex unit combine the activity of the population of simple units? We found that it used readout weights that were proportional to the average interocular receptive field cross-correlation at *near* disparities (Figure S6, red elements; Pearson’s R=0.90, $p<10^{-9}$). In the same manner, the readout weights for the *far* complex unit were proportional to the average interocular receptive field cross-correlation at *far* disparities (Figure S6, blue elements; Pearson’s R=0.89, $p<10^{-9}$). The readout weight is therefore proportional to the interocular receptive field cross-correlation at the preferred disparity of the complex unit.

#### Derivation of the Binocular Likelihood Model

##### Interocular RF Cross-Correlation and Disparity Selectivity

It has been noted elsewhere that computing the cross-correlogram between the left and right receptive fields yields a very good approximation of the disparity tuning curve [11, 64, 65]. Below we present a derivation that describes this relationship. We start by considering the response *r* of binocular simple cells to a given binocular stimulus with disparity δ. The binocular half images (i.e., the images captured by the left and right eyes) are horizontally translated versions of one another. Thus, the stereo pairs presented in a given trial *t* can be defined as $\left\{S_{t}\left(x\right),S_{t}\left(x+\delta \right)\right\}$. As observed experimentally, the response of a binocular simple cell can be well described by linear spatial filtering and rectification, followed by a non-linearity [6, 66],

(13) $$r=g\left({\left[S_{t}\left(x\right)W_{L}\left(x\right)+S_{t}\left(x+\delta \right)W_{R}\left(x\right)\right]}_{+}\right),$$

where $W_{L}\left(x\right)$ and $W_{R}\left(x\right)$ denote the receptive fields of the simple cell for the left and right eyes, and *g* is an expansive nonlinearity. It has been shown that this non-linearity is well described by a power law with an exponent of approximately 2, $g\left(x\right)=x^{2}$, for x>0 [66]. We assume an unrectified squaring non-linearity for mathematical convenience, however, similar results would be obtained for a rectifying squaring non-linearity [65]. Based on this, we can compute a disparity tuning curve, f(δ), by averaging the response of the simple cell across a large number of trials *T*,

$$f\left(\delta \right)=\frac{1}{T}\overset{T}{\sum_{t=1}}r_{t}$$

$$=\frac{1}{T}\overset{T}{\sum_{t=1}}{\left(S_{t}\left(x\right)W_{L}\left(x\right)+S_{t}\left(x+\delta \right)W_{R}\left(x\right)\right)}^{2}$$

(14) $$=\frac{1}{T}\overset{T}{\sum_{t=1}}\left({\left(S_{t}\left(x\right)W_{L}\left(x\right)\right)}^{2}+{\left(S_{t}\left(x+\delta \right)W_{R}\left(x\right)\right)}^{2}+2S_{t}\left(x\right)W_{L}\left(x\right)S_{t}\left(x+\delta \right)W_{R}\left(x\right)\right).$$

As many others have noted [7, 8, 19, 66], the first two terms are monocular and do not depend on binocular disparity – over many trials, these two terms should be a positive constant, *C*, independent of the disparity δ of the stimulus. The disparity dependent modulation of the tuning curve is captured by the interaction term,

(15) $$f\left(\delta \right)=\frac{1}{T}\overset{T}{\sum_{t=1}}2S_{t}\left(x\right)W_{L}\left(x\right)S_{t}\left(x+\delta \right)W_{R}\left(x\right)+C.$$

This expression describes the expected response for a simple cell with receptive fields $W_{L}\left(x\right)$ and $W_{R}\left(x\right)$ to stereoscopic pairs that are translated horizontally in relation to one another by a given disparity δ. Under this formulation, the response of the simple cell is proportional to the stimulus unnormalized cross-correlation, $S_{t}\left(x\right)S_{t}\left(x+\delta \right)$, weighted by the product of the left and right receptive fields, $W_{L}\left(x\right)W_{R}\left(x\right)$, known as the binocular interaction field [66].

However, as we will now show, it is useful to reformulate this expression. Because the stereoscopic pairs are simply translated in relation to the position of the receptive fields, it is equivalent to compute a disparity tuning curve by applying the horizontal shift to the receptive fields, while keeping the stereoscopic images in the same horizontal position (a(x−δ)b(x)=a(x)b(x+δ)),

(16) $$f\left(\delta \right)=\frac{1}{T}\overset{T}{\sum_{t=1}}2S_{t}\left(x\right)W_{L}\left(x\right)S_{t}\left(x\right)W_{R}\left(x-\delta \right)+C$$

(17) $$=\frac{1}{T}\overset{T}{\sum_{t=1}}2S_{t}{\left(x\right)}^{2}\;W_{L}\left(x\right)W_{R}\left(x-\delta \right)+C.$$

Equation 17 is convenient because it expresses the disparity tuning curve as a function of the dot product between the left and right receptive fields, translated according to the disparity δ. This is by definition the cross-correlation between the left and right receptive fields $\left(W_{L}⋆W_{R}\right)\left[\delta \right]$. Note that $\left(1/T\right)\sum_{t=1}^{T}S_{t}{\left(x\right)}^{2}$ is simply the average energy of the stimulus over *T* trials, which influences the amplitude of the tuning curve (but not its morphology). Therefore,

(18) $$f\left(\delta \right)=2\left(W_{L}⋆W_{R}\right)\left[\delta \right]\frac{1}{T}\left(\overset{T}{\sum_{t=1}}S_{t}{\left(x\right)}^{2}\right)+C$$

(19) $$=2\left(W_{L}⋆W_{R}\right)\left[\delta \right]E\left(S_{t}{\left(x\right)}^{2}\right)+C.$$

This formulation provides a mathematically convenient way of expressing tuning for binocular disparity solely based on the receptive fields of simple units. Next, we will take advantage of this convenience to establish a relationship between simple unit properties and their readout by complex units.

##### Optimal Readout of Simple Unit Activity by Disparity Selective Complex Units

In the previous section, we showed that the disparity tuning curve of a simple unit can be well approximated by the scaled cross-correlogram between the left and right receptive fields. We also suggested that stimulus contrast energy induces variability in the firing rate of simple units. This high variability makes simple units unsuitable for the detection of depth. By combining the activities of multiple simple units, complex units provide much better estimates of disparity. The classical disparity energy model obviates this problem by combining the outputs of four simple units with the same preferred binocular disparity, but with their receptive field phase in quadrature [6].

We now ask how could we optimally combine the activities of a population of simple units with highly variable firing rates. Here, we consider not only the variability in firing rate statistics, but also extrinsic variability induced by the stimulus. Inspired by previous work on optimal sensory representations [67], we tackle this problem from a probabilistic viewpoint. Let us interpret the distribution of activity of a simple cell *i* given a particular disparity δ as describing the likelihood of observing the firing rate $r_{i}$ given the disparity δ. We make the simplifying assumption that the response of a simple unit, affected by intrinsic and extrinsic variability, follows a Gaussian distribution around the mean firing rate value, which is given by the corresponding tuning curve, $f_{i}\left(\delta \right)$. Thus, the likelihood for a given simple cell *i* is given by

(20) $$p\left(r_{i}|\delta \right)=\frac{1}{\sqrt{2\pi \sigma_{i}}}e^{-\frac{{\left(r_{i}-f_{i}\left(\delta \right)\right)}^{2}}{2\sigma_{i}^{2}}}.$$

This equation expresses the probability of observing a firing rate $r_{i}$ given a stimulus with disparity δ. Assuming independence across a population of *N* simple cells, we can now combine these probabilities to obtain a joint likelihood,

(21) $$L\left(\delta \right)=p\left(r|\delta \right)=\prod_{i=1}^{N}p\left(r_{i}|\delta \right).$$

By working in log-space, we can convert the logarithm of the product of likelihoods into a sum of logarithms of the likelihood. This is useful because we can express the computation of the likelihood as sum over the activity of many neurons, which is a biologically plausible operation. Equation 21 thus becomes

(22) $$\text{log}L\left(\delta \right)=\overset{N}{\sum_{i=1}}\text{log}p\left(r_{i}|\delta \right)$$

(23) $$=\overset{N}{\sum_{i=1}}\text{log}\left(\frac{1}{\sqrt{2\pi \sigma_{i}}}e^{-\frac{{\left(r_{i}-f_{i}\left(\delta \right)\right)}^{2}}{2\sigma_{i}^{2}}}\right)$$

(24) $$=\overset{N}{\sum_{i=1}}-\frac{{\left(r_{i}-f_{i}\left(\delta \right)\right)}^{2}}{2\sigma_{i}^{2}}-\text{log}\left(\sqrt{2\pi \sigma_{i}}\right)$$

(25) $$=\overset{N}{\sum_{i=1}}\frac{r_{i}f_{i}\left(\delta \right)}{\sigma_{i}^{2}}-\frac{1}{2}\left(\frac{r_{i}^{2}}{\sigma_{i}^{2}}-\frac{f_{i}{\left(\delta \right)}^{2}}{\sigma_{i}^{2}}-\text{log}\left(2\pi \sigma_{i}\right)\right).$$

The second term in Equation 25 can be ignored if we assume that the tuning curves of the population of simple cells cover homogeneously the disparities of interest, and thus $\sum_{i=1}^{N}f_{i}{\left(\delta \right)}^{2}=constant$. Therefore, dropping the quantities that do not depend on the disparity δ, the computation of the log-likelihood simplifies to a sum of the products between the observed simple cell firing rates $r_{i}$, and the corresponding tuning curves, $f_{i}\left(\delta \right)$,

(26) $$\text{log}L\left(\delta \right)=\overset{N}{\sum_{i=1}}r_{i}f_{i}\left(\delta \right).$$

While this is a useful formulation (and technically more generalizable), it is more intuitive to relate readout to binocular correlation. As we observered earlier, the cross-correlogram is a good approximation to the disparity tuning curve of individual simple cells. By replacing $f_{i}\left(\delta \right)$ according to Equation 19 and dropping the constant term that does not depend on disparity, the log-likelihood can be written as

(27) $$\text{log}L\left(\delta \right)=\overset{N}{\sum_{i=1}}r_{i}{\left(W_{L}⋆W_{R}\right)}_{i}\left[\delta \right].$$

Therefore, a population of complex cells can approximate the log-likelihood over disparity simply by weighting the firing rates of individual simple cells by their interocular receptive field cross-correlation. While this particular solution is specific to the assumption of Gaussian variability, the approach followed here could be applied to other forms of response variability using a suitably transformed version of the cross-correlogram. If one assumes Poisson variability, so as to model intrinsic firing rate variability, then the readout form would be a log-transform of the interocular receptive field cross-correlation.

It should be noted that this derivation approximates the behavior of the BNN because Equation 14 used a squaring non-linearity while the BNN used a linear rectification. While this would produce differences in activity, the fundamental response properties are likely to be preserved between this derivation and the BNN.

Finally, we provide an example of a disparity tuning curve obtained using this simple analytical expression. In this simulation, we used 9 simple unit maps with Gabor receptive fields (*f* = 0.0625 cycles/pixel; spatial frequency bandwidth, *b* = 1.5 octaves; σ = 6.27 pixels), covering the full combination of three position disparities ($\text{Δ}x_{0}=\left\{-3,0,3\right\}$ pixels) and three phase disparities (Δϕ={−π,−π/3,π/3} radians). Apart from the number of simple units, we kept the architecture of the model consistent with the Binocular Neural Network. Therefore, the output layer consisted of two complex units – one preferring *near*, the other preferring *far* disparities. The readout weights between simple and complex units were defined according to the analytical expression for our model (Equation 27). This instantiation of the model produced complex units with disparity tuning curves that closely resemble those of complex cells in V1 (Figure S7A): the tuning curves for correlated and anticorrelated stereograms are well approximated by Gabor functions, and anticorrelated tuning curves are inverted and attenuated in relation to correlated stereograms.

The simple units in this instantiation of the model shared the same spatial frequency preference. This demonstrates that our model does not rely on spatial frequency pooling to produce attenuation in response to aRDS. The spatial frequency bandwidth of the output complex unit was smaller than that of the corresponding simple units (1.07 octaves), consistent with the findings that pooling activity across space narrows spatial frequency selectivity [68]. However, our model could also encompass simple units with multiple spatial frequencies, and their activities could be subsequently readout by complex units using the relationship established in Equation 27. In this case, pooling across multiple spatial frequencies would increase the bandwidth of the output complex units, while further reducing the response of the model to spurious disparities [7] and sharpening the degree of disparity selectivity [46, 68].

One prediction stemming from our model is that response saturation in *simple cells* could modulate the amplitude ratio of downstream complex cells. In particular, introducing a compressive nonlinearity at the level of *simple cells*—for instance, to account for sublinear binocular integration [69]—causes the aRDS response to further attenuate relatively to the response to cRDS. We demonstrate this effect in Figure S7B. An expansive non-linearity at the level of simple cells, on the contrary, would cause the degree of attenuation to decrease.

### Quantification and Statistical Analysis

We used bootstrap resampling and we report the corresponding 95% confidence intervals unless otherwise noted. Results were pooled across stimuli or units within a model, but not across different instantiations of models. For the results of fitting procedures, we report the proportion of variance explained by the models.

### Data and Software Availability

We performed all analyses in Python (https://python.org) using standard packages for numeric and scientific computing. The data used for model optimization and implementations of the optimization procedure are available at https://doi.org/10.17863/CAM.8538.

## Author Contributions

N.R.G. conceived the study, implemented the models, performed the analysis, and wrote the manuscript. A.E.W. conceived the study and wrote the manuscript.

## Supplemental Information

Document S1. Figures S1–S7

Document S2. Article plus Supplemental Information
