# Supplementary material for: “What Not” Detectors Help the Brain See in Depth
Source: Curr Biol. 2017 May 22;27(10):1403–1412.e8. doi: 10.1016/j.cub.2017.03.074 (PMC5457481; doi:10.1016/j.cub.2017.03.074)
Supplement: Document S1. Figures S1–S7 [file mmc1.pdf]

Current Biology, Volume *27*

Supplemental Information

``What Not" Detectors Help the Brain See in Depth

Nuno R. Goncalves and Andrew E. Welchman

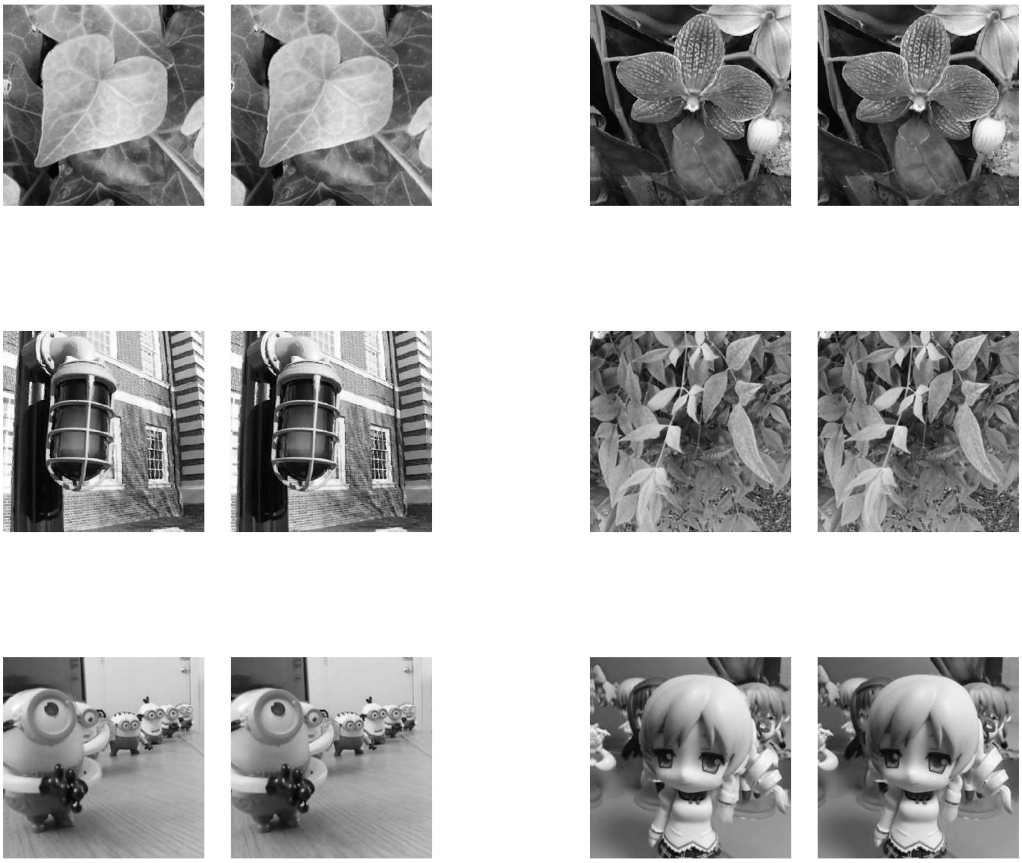

Figure S1: Examples of images used to train the binocular neural network (BNN). Related to Figure 2. Images were extracted from the Light Field Saliency Database [S1], available at http://www.eecis.udel.edu/~nianyi/LFSD.htm. Stereo pairs are rendered for cross fusion.

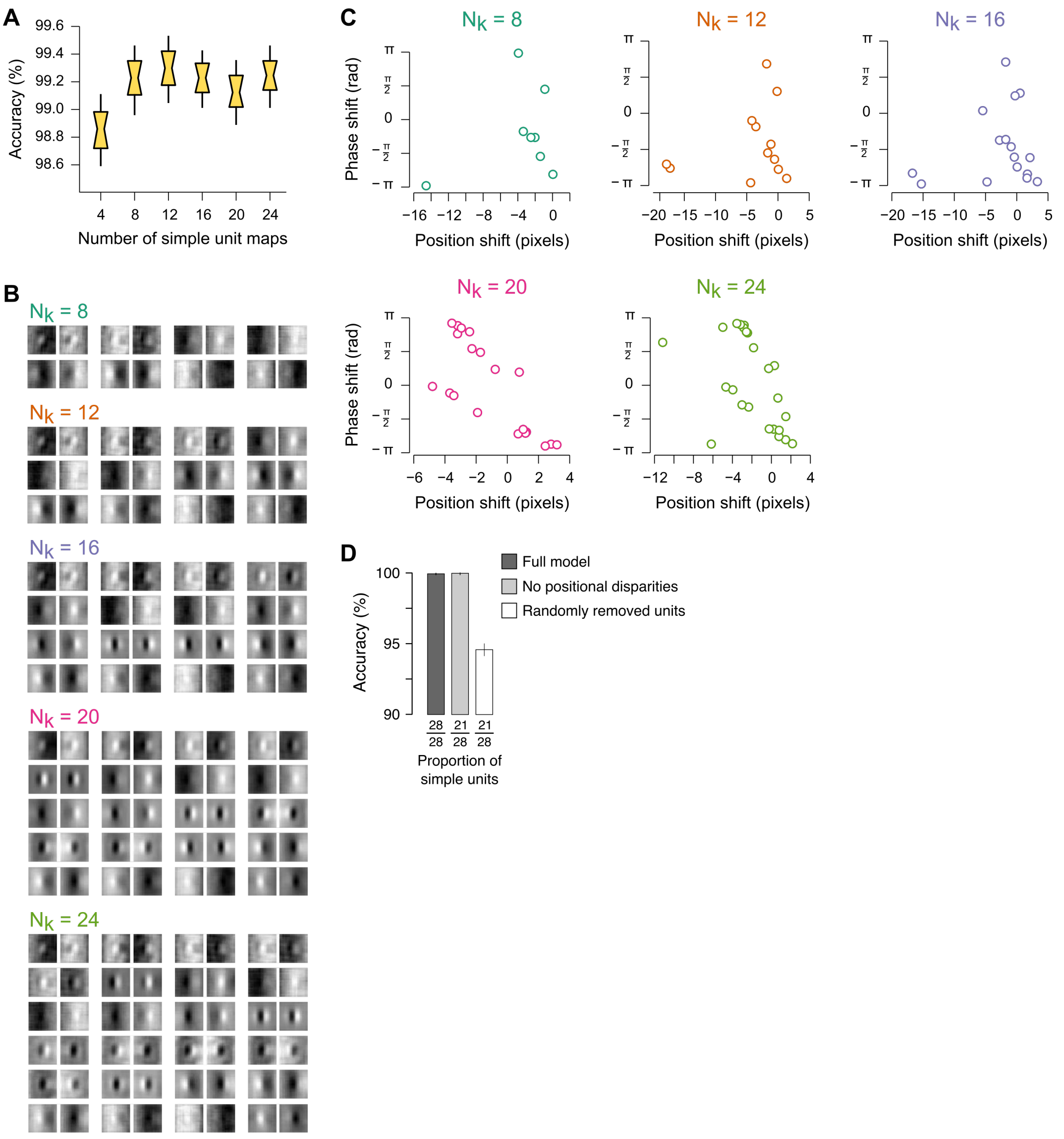

Figure S2: Varying the number of simple units in the network. Related to Figure 2. (A) Decoding accuracy for instantiations of the network with different number of simple units. **(B, C)** Binocular receptive fields developed by the corresponding instantiations, and the respective position and phase disparities. **(D)** Testing the importance of the positional disparity units for the 28 simple unit BNN. Decoding performance is shown for the full model, the model with the positional units removed (i.e., 25% of units around zero phase offsets), and the model with a randomly selected removal of 25% of the non-positional simple units (mean of 1,000 resamples). The limited impact of removing the position disparity units suggest these units do not play a strong role in determining the performance of the BNN.

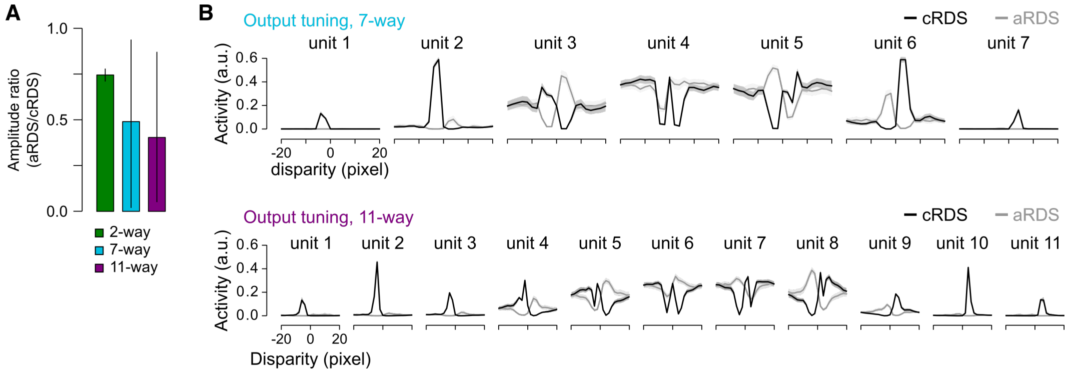

Figure S3: Comparing responses to correlated and anticorrelated stereograms. Related to Figure 3. **(A)** Response attenuation for anticorrelated versus correlated random-dot stereograms for 7- and 11-way classification. Bar graphs depict amplitude ratio (aRDS/cRDS) calculated based on peak-to-peak differences for 2-way, 7-way and 11-way (mean and $CI_{68\%}$ obtained via bootstrapping, 5,000 resamples per output unit). A bias towards values below unity is evident, consistent with the results shown in **Figure 3C**. Note that attenuation appears greater for 7- and 11- way classification. This may result from the network developing more sharply tuned units. **Figure 3C** indicates a hitherto unappreciated difference between the electrophysiological recordings of Cumming & Parker [S2] vs. Samonds et al [S3] at low attenuation ratios. Cumming & Parker [S2] found a bias towards low-amplitude ratios. We speculate that this arose because they sampled closer to the fovea (RFs had eccentricities ranging from 1 to 4 degrees whereas Samonds et al sampled at 4 degrees eccentricity). This difference in sampling strategy may have resulted in recording neurons that were more sharply tuned for disparity, and thus showed greater attenuation for anticorrelated stimuli. **(B)** Disparity tuning curves of output units for 7-way (top) and 11-way classification (bottom). Inversion and attenuation can be observed in the majority of the units. There is considerable variability in the degree of attenuation across units, consistent with neurophysiological observations.

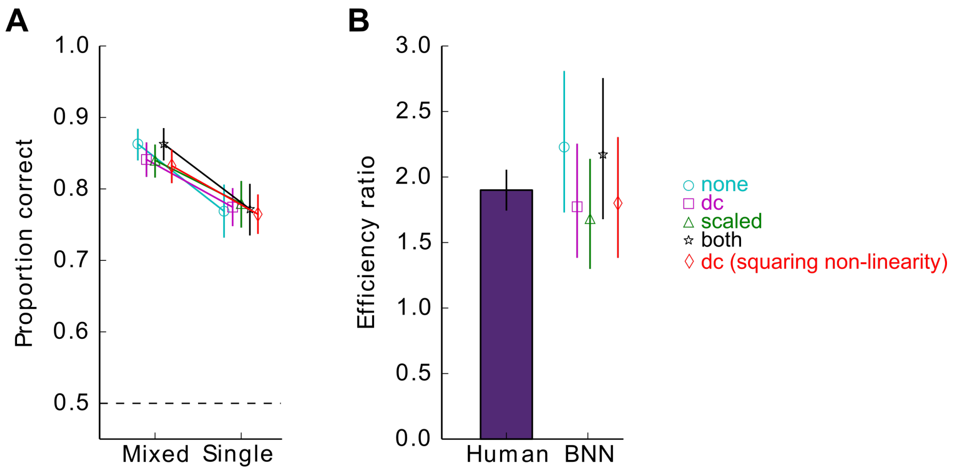

Figure S4: Control analyses for performance on mixed *vs.* single polarity stereograms. Related to Figure 5. We tested the performance of the BNN under several image adjustment conditions: no image adjustment (none); DC correction, in which we subtracted the mean intensity of the input images (dc); scale matching, in which we scaled the images to have the same peak-to-trough range (scaled); and DC correction plus scale matching, in which we removed the mean intensity of the images and scale them to the same peak-to-trough range (both). In the main paper we report results obtained with DC correction, and we re-plot them here to facilitate comparison with the remaining conditions. We ran an additional control in which the rectified-linear nonlinearity was replaced by a (unrectified) squaring nonlinearity **(A)** Proportion of correct trials under the different image adjustment conditions (1,000 trials; error bars show bootstrapped $CI_{95\%}$, 5,000 resamples). **(B)** Efficiency ratios for the BNN (1,000 trials; error bars depict $CI_{95\%}$, 5,000 resamples) plotted alongside experimentally measured efficiency ratios in humans (mean $\pm$ 1 s.d.) [S4].

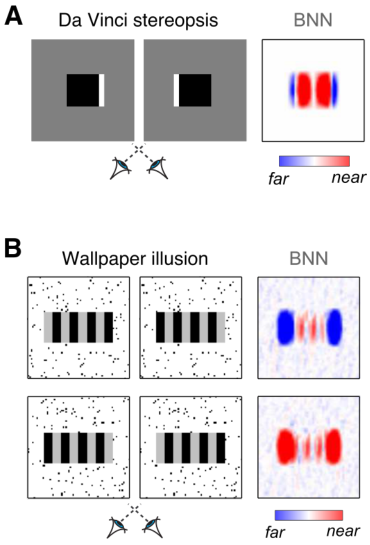

Figure S5: Response of the binocular neural network to half-occluded and wallpaper stimuli. Related to Figure 6. **(A)** Response of the BNN to unpaired bright flanks around a dark occluder. Luminance configuration was inverted relatively to **Figure 6B**. **(B)** Response of the BNN to a wallpaper pattern in which stripes are darker than the background. Compare to **Figure 6C**, where the pattern is defined by bright and dark stripes on a mid-gray background.

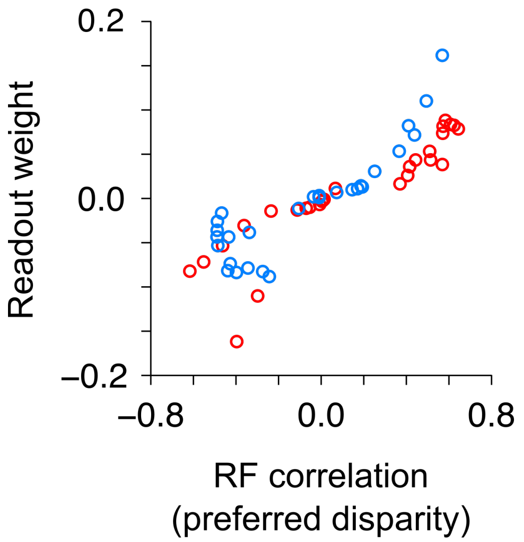

Figure S6: Relationship between the simple unit receptive fields and readout weights. Related to Figure 7. Examining the properties of the BNN showed that the simple unit readout weights are proportional to the receptive field interocular correlation at the preferred disparity. Thus, a complex unit with preferred disparity δ reads the activity of a simple unit with a weight proportional to the correlation between the simple unit's left and right receptive fields at the disparity δ. Red elements: near complex unit; blue elements: far complex unit.

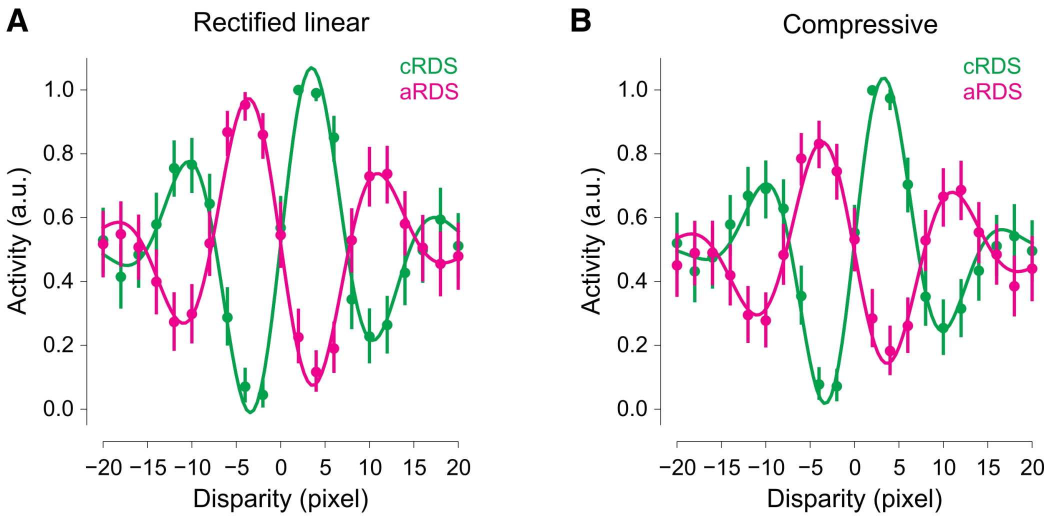

Figure S7: Disparity tuning curves obtained in a simple instantiation of the Binocular Likelihood Model (BLM). Related to Figure 7. Tuning curves were computed for correlated (green elements) and anticorrelated (pink elements) stereograms. Solid lines represent Gabor fits. Error bars depict resampled $CI_{95\%}$ (5,000 resamples). **(A)** Tuning curves for cRDS and aRDS assuming simple units with linear rectification **(B)** As in **A**, but assuming simple units with a compressive non-linear activation function (in this case, the square root) - i.e. effectively implementing sublinear binocular integration [S5]

Supplemental References

[S1] Li, N., Ye, J., Ji, Y., Ling, H., and Yu, J. (2014). Saliency Detection on Light Field. IEEE Conference on Computer Vision and Pattern Recognition , 2806–2813.

[S2] Cumming, B.G. and Parker, A.J. (1997). Responses of primary visual cortical neurons to binocular disparity without depth perception. Nature *389*, 280–3.

[S3] Samonds, J.M., Potetz, B.R., Tyler, C.W., and Lee, T.S. (2013). Recurrent connectivity can account for the dynamics of disparity processing in V1. J Neurosci *33*, 2934–46.

[S4] Harris, J.M. and Parker, A.J. (1995). Independent neural mechanisms for bright and dark information in binocular stereopsis. Nature *374*, 808–811.

[S5] Longordo, F., To, M.S., Ikeda, K., and Stuart, G.J. (2013). Sublinear integration underlies binocular processing in primary visual cortex. Nature neuroscience *16*, 714–723.
